# Hierarchical flow of sensory information in rat somatosensory cortex

**DOI:** 10.1186/1471-2202-15-S1-P172

**Published:** 2014-07-21

**Authors:** Houman Safaai, Yanfang Zuo, Miguel Maravall, Stefano Panzeri, Mathew E Diamond

**Affiliations:** 1 Tactile Perception and Learning Laboratory, International School for Advanced Studies (SISSA), 34136 Trieste, Italy; 2 Center for Neuroscience and Cognitive Systems, Istituto Italiano di Tecnologia, Corso Bettini 31, 38068, Rovereto, Italy; 3 Instituto de Neurociencias de Alicante, Consejo Superior de Investigaciones Científicas-Universidad Miguel Hernández, 03550 Sant Joan d'Alacant, Spain

## 

Through intracortical processing, sensory stimuli are encoded initially according to elemental features and finally by the action to be taken as a result of the stimulus. The transformation between cortical regions is still not understood although it is known that the participation of primary somatosensory cortex (S1) and secondary somatosensory cortex (S2) differs according to behavioral task [1]. To investigate the sensory information flow between S1 and S2, we recorded from both areas as rats identified textured plates through whisker palpation [2]. Neurons in S2 typically fired with a longer latency to whisker contact, consistent with sequential processing. We used linear discriminant analysis of firing rates of populations of simultaneously recorded neurons [3] in S1 and S2 to compare how they encoded the stimulus. Populations in both regions reliably encoded the stimuli, but the discriminability afforded by neuronal populations in S2 reached a peak 50 ms later than that of S1 populations, also consistent with sequential processing. Different rats were trained with different combinations of texture/reward associations from a set of three textures and two reward locations (left and right). This made it possible to compare the decoding of texture and the decoding of the choice of the animal (left turn versus right), both in correct and incorrect trials. S1 populations carried more information about texture while S2 populations carried more information about choice. Using linear classifiers based on the texture and turn direction classes, we compared the trial-by-trial relationship of the stimulus representation in the two regions to the animal’s choice. When neuronal populations in both regions correctly encoded the stimulus, the rat made the correct choice with 85% probability. When neuronal populations in both regions incorrectly encoded the stimulus, the rat made the correct choice with just 30% probability. When S1 and S2 had disparate representations of the stimulus, the choice of the rat was more than twice as likely to be consistent with the output of S2 than with S1. Thus, neuronal activity in S2 appeared to have a more direct connection to the rat’s decision. The above findings suggest a causal flow of signals from S1 to S2; to test this idea, we computed the directed information [4] between single S1 and S2 units as well as between the S1 and S2 population vectors. We found a significantly higher causality index for the texture information flow from S1 to S2 as the rat palpated the texture, while the sign and value of the causality changed during the decision period when the rat already made its decision. Finally, using a linear feedforward neural network trained with S1 or S2 inputs and outputs, and tested with S1 or S2 inputs and outputs, we found that the feedforward network with S1 input and S2 output had significantly higher performance with respect to the reverse directionality of the input and output. All these findings suggest a hierarchical flow of information between cortical regions, as sensation is transformed to percept and choice.
